# Bioprinting of Stem Cells in Multimaterial Scaffolds and Their Applications in Bone Tissue Engineering

**DOI:** 10.3390/s21227477

**Published:** 2021-11-10

**Authors:** Shebin Tharakan, Shams Khondkar, Azhar Ilyas

**Affiliations:** 1 Bio-Nanotechnology and Biomaterials (BNB) Lab, New York Institute of Technology, Old Westbury, NY 11568, USA; stharaka@nyit.edu (S.T.); skhondka@nyit.edu (S.K.); 2 New York Institute of Technology, College of Osteopathic Medicine, Old Westbury, NY 11568, USA; 3 Department of Bioengineering, New York Institute of Technology, Old Westbury, NY 11568, USA; 4 Department of Electrical and Computer Engineering, New York Institute of Technology, Old Westbury, NY 11568, USA

**Keywords:** bioprinting, stem cells, composite biomaterials, osteogenesis, fracture repair

## Abstract

Bioprinting stem cells into three-dimensional (3D) scaffolds has emerged as a new avenue for regenerative medicine, bone tissue engineering, and biosensor manufacturing in recent years. Mesenchymal stem cells, such as adipose-derived and bone-marrow-derived stem cells, are capable of multipotent differentiation in a 3D culture. The use of different printing methods results in varying effects on the bioprinted stem cells with the appearance of no general adverse effects. Specifically, extrusion, inkjet, and laser-assisted bioprinting are three methods that impact stem cell viability, proliferation, and differentiation potential. Each printing method confers advantages and disadvantages that directly influence cellular behavior. Additionally, the acquisition of 3D bioprinters has become more prominent with innovative technology and affordability. With accessible technology, custom 3D bioprinters with capabilities to print high-performance bioinks are used for biosensor fabrication. Such 3D printed biosensors are used to control conductivity and electrical transmission in physiological environments. Once printed, the scaffolds containing the aforementioned stem cells have a significant impact on cellular behavior and differentiation. Natural polymer hydrogels and natural composites can impact osteogenic differentiation with some inducing chondrogenesis. Further studies have shown enhanced osteogenesis using cell-laden scaffolds in vivo. Furthermore, selective use of biomaterials can directly influence cell fate and the quantity of osteogenesis. This review evaluates the impact of extrusion, inkjet, and laser-assisted bioprinting on adipose-derived and bone-marrow-derived stem cells along with the effect of incorporating these stem cells into natural and composite biomaterials.

## 1. Introduction

Bone fractures in the United States are projected to increase 50% by 2025. Individuals in the age group 65 to 74 are estimated to have the fastest increase of 87% [1]. The increasing rate of fractures warrants novel and innovative methods of treatment. Autografts and allografts are the standard clinical solutions, with their respective advantages and disadvantages. Bone autografts are continuously considered the orthopedic gold standard in bone tissue transplantations. Autologous bone grafts are commonly taken from the iliac crest with donor site morbidity associated with the transplant [2]. New studies, however, are examining the potential efficacy of proximal tibial grafts due to lower post-operative pain and similar healing properties to the iliac crest [3,4]. Autografts are resorbable, osteoconductive, osteoinductive, and provide a living source of cells [5]. Donor site morbidity and infection are common concerns regarding autografts [6]. Unlike autografts, allografts require the graft from a cadaver or another individual. Cadavers lack living osteoblasts and osteoprogenitors, thereby limiting the osteogenic potential of the graft [7,8]. Additionally, allografts are associated with a greater risk of an immune response and graft rejection but have lower donor site morbidity [5]. Apart from autologous or allograft bone transplantations, synthetic or natural polymeric materials may be used in their place. 

Synthetic or natural polymers may be used in the place of autografts. These polymers need to be comparable to autografts, and with fewer disadvantages, in order to be used effectively in a clinical setting [9]. The assembly of synthetic or natural materials into usable and implantable constructs can be achieved with 3D bioprinting to create scaffolds. 3D bioprinting is an extension of additive manufacturing. 3D bioprinting, by definition, is the printing of constructs with the incorporation of viable cells, biomaterials, or biological materials [10,11]. The initial chemical mixture is referred to as the bioink, and once bioprinted it becomes known as a scaffold or construct, and can be implanted. With recent advances in 3D bioprinting technology, biomimetic scaffolds are used to accelerate bone regeneration in vitro and in vivo using hydrogels or composite structures. Hydrogels are a gel-like macromolecular complex creating a 3D network of polymers, with polymers such as collagen, hyaluronic acid, or alginate. Bone is traditionally considered dense solid tissue, with the principal component being hydroxyapatite. Thus, repair of bone can be associated with metal implants such as titanium [12]. In contrast, hydrogels are soft gel-like constructs that can be directly applied to bone to induce regeneration with the inclusion of cells and growth factors. The efficacy of soft materials has been shown through in vivo calvaria and femoral defect models, while clinically a mix of both soft and hard materials such as metals can be used. Additionally, hydrogels can be 3D bioprinted as composites, meaning that they can contain multiple polymers intertwined to influence the physiochemical properties of the printed scaffold. The hydrogel scaffolds implement natural or synthetic biomaterials to form porous constructs to repair fractures in place of an autograft [13]. An ideal scaffold is biocompatible, biodegradable, osteoconductive, osteoinductive, and can resist compressive forces. Natural materials include proteins such as collagen, silk, and fibrin and polysaccharides such as alginate, chitosan, and hyaluronic acid. Natural polymers allow cell incorporation with high cell viability but have disadvantages such as low mechanical strength and varying biodegradability. The use of these materials creates natural scaffolds once the bioink has undergone 3D bioprinting. Natural polymers tend to have high cell adhesion due to the presence of cell adhesion molecules such as integrins [14,15]. A notable exception is alginate which lacks these binding sites for cells. This is resolved by functionalizing alginate with a RGD peptide sequence to permit cell adhesion [16]. Synthetic polymers are the opposite with increased mechanical strength and greater fine-tuning of biodegradability, forming synthetic scaffolds [17]. Unlike natural polymers, synthetic materials are lacking in bioactivity [18]. Included in this class of polymers are poly(ε-caprolactone) (PCL), poly(lactide-co-glycolide) (PLGA), and polyglycolic acid (PGA) [19]. Alternatively, functionalized polymers can be used in place to demonstrate similar efficacy. Multiple materials can be combined to form a composite scaffold which assimilates the properties of each polymer. These combinations confer improved biocompatibility, biodegradation, and mechanical properties to improve the desired parameters [20,21,22]. 

The hydrogel constructs can be printed with cells such as osteoblasts or metal ions to further speed up the healing process [21,22]. Alternatively, mesenchymal stem cells can be used in place of osteoprogenitor or osteoblast cells. Mesenchymal stem cells (MSCs) are multipotent and can differentiate into cartilage, bone, adipose, muscle and other tissues depending on the growth factors present making them versatile and useful for tissue engineering. Human mesenchymal stem cells (hMSCs) were initially harvested from bone marrow, but have now been isolated from adipose tissue, amniotic fluid, placental tissue, Wharton’s jelly, endometrium, and dental pulp [23,24]. Li et al. evaluated the osteogenicity of hMSCs and found Wharton’s Jelly MSCs to have the greatest osteogenic potential, followed by placental, adipose, and bone marrow stem cells [25]. Adipose and bone marrow stem cells both have similar osteogenic capabilities, but different disadvantages with their use. Adipose-derived stem cells (ADSC) are easy to harvest, but require more testing to evaluate their capabilities in bone regeneration, while bone marrow stem cells (BMSC) are extracted in low quantities and require extensive culturing [26]. Coupled with 3D bioprinting, these cells provide osteoinductive capabilities which improve bone regeneration [27]. A benefit of 3D bioprinting with stem cells or cell-lines is the incorporation of cells directly into the bioink for immediate printing. Compared to seeding cells post printing, 3D bioprinting cells in conjunction with the biomaterials offers a streamlined process to generate multiple samples without the waiting time for cell attachment by seeding. A significant advantage, however, is the homogenous distribution of cells during the printing process, which may not be conferred during cell seeding. Homogenous dispersion provides the benefit of a functional culture that can increase the formation of tissue [28]. However, cell viability must be confirmed post printing due to pressure differentials and stress during the printing process. Aside from cell stress, some 3D bioprinters are expensive and may not be academically available. 

3D bioprinters are multifaceted and can have uses in different fields, ranging from tissue engineering to biosensor manufacturing. In particular, 3D bioprinters are capable of printing high-performance bioink for biosensor applications [29]. High-performance bioinks are next-generation bioinks with reinforcement mechanisms to drive cell functions [30]. The functionality of biosensors involves appropriate conductivity and electrical transmission. Organ-wise, this applies to cardiac tissue due to electrical conduction through intercalated discs. In contrast to bone tissue, electrical conductivity is not a primary concern for fracture studies. A recent study on cardiac tissue regeneration involved the use of gold nanocomposite bioinks to improve cardiomyocyte and cardiac fibroblast functionality [31]. The functional use of this bioink can be further developed to create biosensors and medical devices to assist cardiac function. Furthermore, to demonstrate the diverse potential of 3D bioprinting, a separate study devised a high-performance alginate bioink with conductive silver nanoparticles and chondrocytes to synthesize a bionic ear [32]. The potential to use 3D bioprinting to create electrical configurations and biosensors is untold and can only expand in the future. This paper will expand on three 3D bioprinting techniques and their impact on the viability, differentiation, and osteogenicity of adipose-derived and bone marrow stem cells. The osteogenic effects of both stem cells will be evaluated in hydrogel and composite biomaterial scaffolds to determine their efficacy in bone fracture repair. 

## 2. Bioprinting Techniques

### 2.1. Extrusion Bioprinting

**Figure 1:**
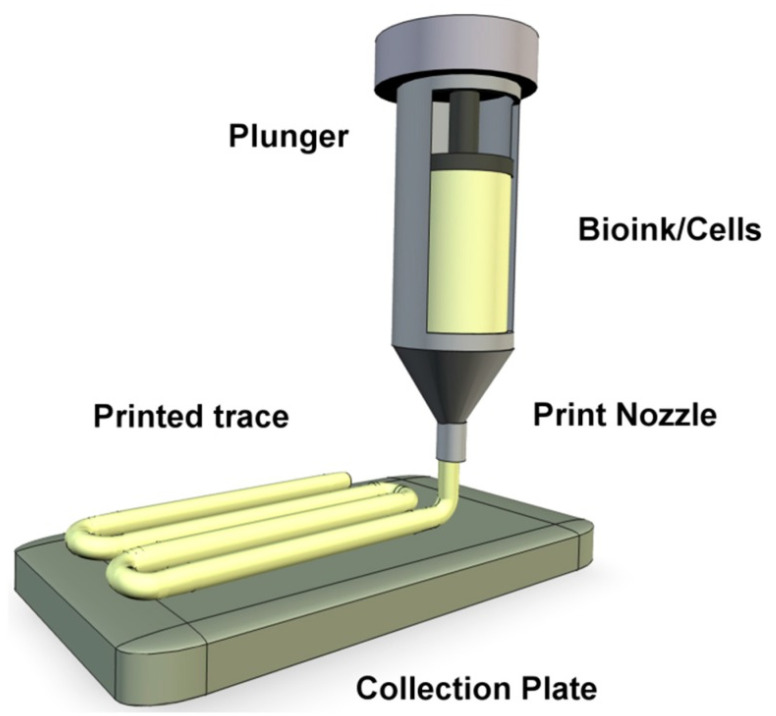
A representation of extrusion bioprinting of bioink onto a collection plate as a continuous filament [38].

Extrusion bioprinting is cost effective and is the most used method in 3D bioprinting. Extrusion bioprinters can contain multiple printheads to mix many materials together providing various 3D structures comprising of different cells, biomaterials, and signaling molecules [33]. In this method, cartridges are loaded with the biomaterials or bioink and continuous filaments are printed directly onto a stage (Figure 1). Continuous bioink filaments instead of droplets are extruded through the printer due to the continuous force applied. The extrusion mechanism can be pneumatic, piston, or screw driven, with temperature or pressure being computer controlled [34]. Previous studies have indicated the range of viscosity for printable biomaterials is between 30–6 × 10^7^ mPa·s indicating the extrusion method can print very viscous bioinks. The printing resolution is poor, with the range being between 200–1000 µm, compared to other methods. Extrusion fabrication speed is the slowest, and is between 10 µm s^−1^–700 mm s^−1^ [35]. Current extrusion bioprinters are capable of printing high cell concentrations, including cell spheroids, but are limited by lower cell viability due to the high extrusion and shear pressure [36,37].

#### 2.1.1. Extrusion Bioprinting of Adipose-Derived Stem Cells

Shear forces have been shown to impact differentiation towards endothelial and bone lineage [39]. Zhang et al. exposed human ADSCs (hADSC) to 12 dynes of shear stress, which stimulated endothelial differentiation and acquisition of a nonthrombogenic phenotype [40]. Colle et al. printed spheroid ADSCs onto GelMA scaffolds and demonstrated cell viability was equal at strut edges and centers. After 7 days of culturing the GelMA-encapsulated spheroids, the viability of the 3D printed scaffolds was 79%, while non-printed had 80% viability. The printed scaffolds and non-printed scaffolds had no significant difference in viability [41]. Interestingly, Leucht et al. created and printed a bone vascularization bioink with hADSCs and human dermal microvascular endothelial cells (HDMECs). The co-cultured hydrogels consisted of the osteogenic hADSCs and vascular compartment (hADSCs + HDMECs). The cells were viable after being printed and increased levels of Col I, fibronectin, ALP, and OPN at 20 days in the co-culture demonstrated osteogenic differentiation [42]. Wang et al. showed that cell viability on day 1 after printing was 88.13%, with viability increasing to 90.41% 7 days later. The increased expression of vinculin 7 days after printing showed that cells could attach to the hydrogels and grow normally. Blood vessel ingrowth and bone matrix formation were observed after 8 weeks of implantation in vivo, showing osteogenic capacity of hADSC in forming new bone [43]. The hADSCs pre-differentiated for 3 weeks and printed in a Fibrin/Gelatin/Hyaluronic Acid/Glycerol hydrogel demonstrated greater hydrogel calcification than non-differentiated hADSCs. 3D-printed hADSC monolayers displayed high viability 2 days after printing, with 97.1% of the cells surviving [44]. Extrusion bioprinting ADSCs provides adequate viability, proliferation, and retention of differentiation ability. The effect of extrusion printing on adipose-derived stem cells is an avenue for investigation for bone repair as it has demonstrated potential capabilities in osteogenesis.

#### 2.1.2. Extrusion Bioprinting of Bone-Marrow-Derived Stem Cells

The high shear stress from extrusion bioprinting can induce cells into a certain lineage. Yourek et al. determined the effects of fluid shear stress on human BMSCs (hBMSC). The findings suggested that shear stress encourages differentiation into the osteoblast lineage. The upregulation of BMP-2, Bone sialoprotein, and Osteopontin after 4 days indicates that shear stress encourages osteogenic gene expression [45]. However, goat BMSCs (gBMSC) bioprinted by Fedorovich et al. expressed ALP after 2 weeks of culturing in osteogenic media, implying that stem cells retain their long-term differentiation potential. It was determined that needle diameter had no significant effect on cell viability 5 h after deposition. Bioprinting had no adverse effect on the gBMSCs, but hydrogel composition impacted cell viability. Matrigel and alginate scaffolds were shown to have greater cell survival after 7 days compared to agarose and Lutrol F127 scaffolds [46]. Rat BMSC (rBMSC) microbeads printed by pneumatic extrusion appeared morphologically round and were evenly distributed throughout the alginate dialdehyde-gelatin (ADA-GEL) and nano-scale glass bead (ADA-GEL-nBG) scaffold. Pneumatic pressure changes from 2.3 to 2.5 bars during microbead printing had no impact on rBMSC survivability. Cell viability was 85% for the ADA-GEL scaffold and 75% for the ADA-GEL-nBG after 7 days of culturing, implying that the materials are not cytotoxic [47]. The lower viability may be due to the pneumatic extrusion printing as this method places pressure on the cells. Du et al. printed methacrylamide gelatin scaffolds with rBMSCs and determined cell viability and DNA content after mechanical extrusion. Cell viability was 91.8%, but increased to 94.9% on day 28. After extrusion, DNA content was less than 30%, showing immediate low proliferation. However, by day 28, DNA content increased to almost 70%, indicating proper proliferation [48]. Finally, extrusion bioprinting may have effects on cell survivability and stemness due to the high shear pressure but generally, no adverse effects appear to occur.

### 2.2. Inkjet Bioprinting

**Figure 2:**
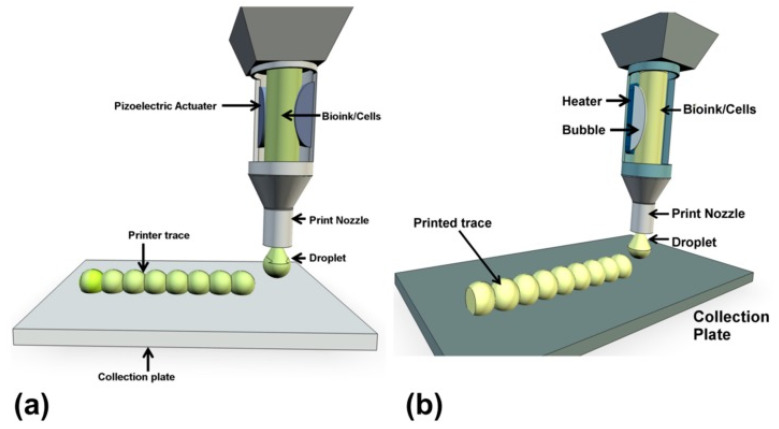
A representation of inkjet bioprinting of droplets onto collection plates with a piezoelectric actuator (**a**) and a thermal actuator (**b**) [38].

Unlike extrusion bioprinting, inkjet bioprinting employs discrete droplets as the primary structural component in 3D constructs deposited onto a collection plate. A thermal or piezoelectric actuator is used to generate droplets of the desired size by creating pressure increases to cause propulsion of the bioink (Figure 2). Inkjet bioprinting can be classified as continuous inkjet (CIJ) bioprinting or drop-on-demand (DOD) bioprinting, with DOD being the most suitable option for tissue engineering [49]. The use of inkjet bioprinting confers high printing precision, along with low cost and accessibility [38,49]. However, the incorporation of viscous cell-laden bioinks can damage the nozzle due to clogging, which in turn hinders cell viability and function. It has been reported that the printable viscosity is less than 10 mPa·s. Additionally, this printing method has a high resolution of between 10 and 50 µm, and a fast fabrication speed of 10^5^ droplets/s [29,35]. The downside of this method is the inability to print high cell concentrations, since higher cell densities increase bioink viscosity [50]. The reported cell densities are less than 10^6^ cells/mL, limiting the potential for bioprinting with highly viscous biomaterials [35].

#### 2.2.1. Inkjet Bioprinting of Adipose-Derived Stem Cells

Kim et al. used piezoelectric inkjet printing to construct an hADSC-laden PLGA scaffold on polystyrene substrate. The PLGA inks were prepared by dissolving PLGA in N,N-dimethylformamide followed by 0.2-micron filtration for piezoelectric inkjet printing. The PLGA printed patterns were favorable for hADSC adhesion, with adhesion >20%, but the polystyrene was less than 10% 24 h after printing. The choice of biomaterial impacts immediate cell adhesion, as demonstrated with the greater PLGA cell adhesion. At 24 h after printing, cell proliferation was 154%. Proliferation rate increased 275% after 72 h post printing, indicating that the hADSCs retained their proliferative capabilities. Since inkjet printers are limited to printing lower cell densities, the PLGA patterns were incomplete, with the hADSCs resulting in partially filled constructs. The variable patterns created by inkjet printing are simple and suitable for analyzing geometrical effects on hADSC or stem cell behavior [51]. 

More studies should be conducted on ADSC phenotype expression, differentiation potential, and viability immediately after inkjet printing. The effect of the thermal or piezoelectric actuators on ADSC survivability should be evaluated in-depth. Currently, ADSCs can differentiate into osteoblasts with the help of osteogenic differentiation media under hydrostatic pressure [52]. It has also been shown that ADSCs are able to lean toward a chondrogenic phenotype with no exposure to chondrogenic soluble factors under hydrostatic pressure [53]. The effect of inkjet pressure should be explored further to evaluate the differentiation ability of ADSCs into the osteoblast lineage. 

#### 2.2.2. Inkjet Bioprinting of Bone-Marrow-Derived Stem Cells

Blaeser et al. extracted hMSCs from the femoral head of five donors and evaluated shear stress in cell-laden alginate scaffolds. Cell proliferation and viability were not significantly affected at low shear pressures (<5 kPa), but were strongly affected at higher shear pressures (>10 kPa). Interestingly, medium shear pressures (5–10 kPa) encouraged cell proliferation, indicating that moderate shear pressure has stimulatory effects [37]. Previously, high mechanical pressure has been shown to differentiate mesenchymal stem cells towards an osteoblast lineage [54]. However, the stem cell phenotype remained unchanged post printing, with the detection of vimentin, a surface marker in mesenchymal stem cells. The pressure threshold is near 5 kPa for cells to be printed without side-effects [37]. Gao et al. inkjet bioprinted acrylated peptides and PEG hydrogels with hBMSCs and determined that cell viability after 24 h was 87.9%, indicating that cells were preserved post printing. Osteogenic differentiation did not seem to be affected by printing; however, RUNX2 expression was consistently elevated in the PEG-peptide scaffold, indicating long-term osteogenic differentiation. ALP levels were markedly increased by day 7, showing accelerated osteoblast formation [55]. hBMSCs printed in PEG-GelMA scaffolds exhibited viability greater than 80% immediately after printing. The inkjet printing permitted the formation of evenly distributed cells in a layer-by-layer fashion for bone tissue synthesis. Unfortunately, GelMA is highly viscous, which hinders printability. Moreover, the scaffolds were simultaneously photopolymerized, which had no significant negative effects on the hBMSCs [56]. 

Since this method of printing results in low resolution and cell concentrations, BMSCs should be printed in low concentrations to avoid nozzle clogging and adverse cell effects. Optimal bioinks should be developed to maximize BMSC viability, proliferation, and differentiation to increase their osteogenic effects. Additionally, more studies should be conducted to evaluate the effects of the inkjet actuators (thermal vs. piezoelectric) on BMSC stemness, osteogenesis, and viability. 

### 2.3. Laser-Assisted Bioprinting

**Figure 3:**
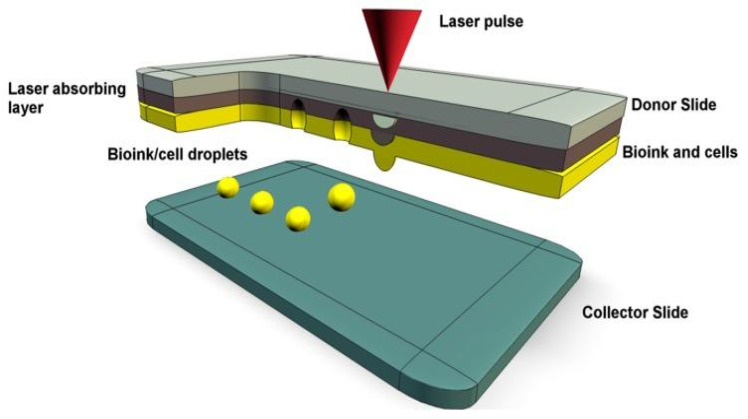
A representation of laser-assisted bioprinting with bioink droplets deposited by laser pulses onto a collector slide [38].

Laser-assisted bioprinting, or laser-induced forward transfer (LIFT), uses droplet release like the inkjet bioprinting system. A laser pulse encounters the top donor layer, which forms a bubble to propel the bottom bioink layer as droplets onto the collection plate (Figure 3) [57]. Unlike extrusion and inkjet bioprinting, there is no contact with a nozzle, which eliminates the possibility of clogging and shear pressure. Due to this, cell viability is higher compared to the other two methods (>95%) and viscous bioinks are printable [58]. Printable bioink cell densities are less than 10^8^ cells/mL, with viscosity between 1 and 300 mPa·s [35]. The greatest strength of laser-assisted bioprinting is the high printing speed and precision, which allows for fine-tuned 3D structures capable of mimicking natural tissues [34]. Printing resolution is reported to be between 10 and 100 µm, with fabrication speed being 200–1600 mm s^−1^ [35]. This method is the most expensive and complex, which limits its use commercially [38]. However, LIFT is used much more often in bioprinting and offers potential in printing stem cells due to the capability of creating complex 3D structures. A comparison of each printing method is summarized in Table 1, which is adapted from [35]. 

#### 2.3.1. Laser-Assisted Bioprinting of Adipose-Derived Stem Cells

Koch et al. determined that laser-assisted bioprinting did not initiate differentiation due to conservation of the hADSC immunophenotypes CD44, CD105, CD29, and CD90 [59]. The viability of hADSCs post printing was determined to be 99.7% in one study conducted. Furthermore, the proliferative ability of the stem cells was shown to be unimpacted. DNA damage in printed hADSCs was not significant relative to the control cells showing an absence of genotoxicity indicating laser exposure may not have adverse effects [60]. A study fabricating corneal tissue with hADSCs in human Col I hydrogels showed high viability immediately after printing with a Nd:YAG and Er:YAG laser. The hADSCs retained proliferative capabilities as Ki67 was expressed on day 1 and day 4 post printing. The proliferation rate significantly increased after 4 days of culturing which may be due to the biocompatible nature of collagen [61,62]. A separate study conducted by Gruene et al. laser printed hADSCs in alginate/EDTA blood plasma hydrogels to evaluate the effects of laser printing. There was no change in cell behavior, which was determined by measuring cell proliferation, which showed no significant difference in the laser printed cells. The cells survived the stress of the laser printing and retained their differentiation potential into adipocytes, which was verified by Oil red O staining and RT-qPCR for adipogenic genes [63]. 

Overall, laser printing hADSCs has no detrimental effect on their proliferation, viability, and differentiation, making it an optimal printing method for creating cell-laden scaffolds. However, there may be unwanted differentiation due to the physical forces present during the printing process [64]. More studies should be conducted to determine the effects of laser exposure in the manner of osteogenic differentiation of ADSCs.

#### 2.3.2. Laser-Assisted Bioprinting of Bone-Marrow-Derived Stem Cells

Gruene et al. printed porcine BMSC (pBMSC) hydrogel scaffolds through laser-assisted bioprinting and determined cell viability and differentiation potential post printing. Cell viability and proliferation exhibited no significant difference, and no changes were detected in pBMSC phenotype. The pBMSCs displayed an increase in aggrecan expression with a lack of collagen type II expression. The findings indicated that MSCs in a scaffold are predisposed to shift to chondrogenic differentiation in a 3D culture. Additionally, it was determined that laser bioprinting caused no significant spontaneous differentiation into osteoblasts by measuring ALP activity [65]. A separate study conducted by Koch et al. determined that there was no significant difference in apoptosis, proliferation, and genotoxicity in hBMSCs post printing with a Nd:YAG-laser. The hBMSCs demonstrated a survival rate of 90% after printing [59]. Laser bioprinting confers printing with high resolution and precision, as shown by a printing resolution of 138 µm and precision of 16 µm in one study with BMSCs [66]. Ali et al. used slow jet conditions, which are more stable, to minimize droplet impact energy with mice BMSCs (mBMSC). The slow jetting conditions have decreased laser pulse energy, which reduces shear stress. The mBMSCs were printed with high cell viability, which was measured 24 h after printing, and possessed high resolution [67]. Laser bioprinting can be used to deposit BMSCs directly in vivo to enhance osteogenesis. Keriquel et al. devised a method to print nano-Hydroxyapatite (nHA) layers directly onto a mouse critical sized calvaria defect. The experimentation demonstrated laser exposure to the dura mater caused temporary inflammation and no permanent tissue damage in mouse brain [68]. This was further expanded by printing BMSCs in situ in a ring or disk geometry to induce osteogenesis in vivo. The in situ printed BMSC nHA disks showed significant osteogenesis than the ring shaped BMSC nHA. It is hypothesized that due to the disk cell homogeny and proximity, the BMSCs secreted paracrine factors to induce osteogenic differentiation [69]. This novel technique should be explored in greater depth with different biomaterials and BMSCs to gauge its full potential. A summary of each bioprinting technique and its effects on ADSCs and BMSCs is provided in Table 2.

**Table 1:** Bioprinting techniques.

	Extrusion [50,70,71,72,73,74,75,76,77,78,79,80]	Inkjet [50,51,80,81,82,83,84,85,86]	Laser Assisted [50,75,80,87,88]
Viscosity of the Bioink	30–6 × 10^7^ mPa·s	<10 mPa·s	1–300 mPa·s
Cell Density	High, cell spheroids	Low, <10^6^ cells/ml	Medium (10^8^ cells/mL)
Resolution	200–10^3^ µm	10–50 µm	10–100 µm
Speed of Fabrication	10–700 mm/s	10^5^ droplets/s	200–1600 mm/s
Cell Viability	80–90%	>85%	>95%
Price	Moderate	Low	High
Advantages	High-viscosity printing, print high cell densities	Inexpensive, high printing speed, moderate cell viability	High printing speed and precision, high cell viability
Disadvantages	High shear stress, lower cell viability, slow printing	Low cell density, low viscosity biomaterials, nozzle clogging	Expensive, complex laser control

## 3. Scaffolds

Prior to 3D bioprinting, a bioink must be created. Bioink is referred to as the mixture of cells and biopolymers or biomaterials. The properties of the bioink vary and are largely dependent on the ink’s constituents. Synthetic or natural materials may be used to create synthetic or natural bioink. Once the bioink has undergone the bioprinting process, a scaffold is formed. Scaffolds are the structural form of a bioink and provide micro and macro-architecture for cell attachment in vitro and for in vivo implantation. In this paper, we define “scaffolds” as multipurpose with the function of being tested in vitro or in vivo. The following sections evaluate the use of scaffolds with varying bioink constituents in bone tissue regeneration.

### 3.1. Adipose-Derived Stem Cells

#### 3.1.1. Natural Scaffolds

##### Alginate

Jia et al. bioprinted four RGD-alginate hydrogels with varying oxidation and concentrations with hADSCs in a lattice structure through the use of a custom inkjet printer. The alginate was oxidized with the addition of sodium periodate at room temperature. However, crosslinking was performed with the addition of a 100 mM CaCl_2_ gelatin substrate. The substitution of sodium ions for calcium ions allows the strengthening of the polymer yielding a solid hydrogel. Post cross-linking, the rigid form of the alginate hydrogel allows it to resist compressive forces. Alginate with 5% oxidation and 15% concentration had the greatest hADSC proliferation and spreading compared to the 0% oxidized and 8% concentration alginate scaffold. This was potentially due to the greater porosity and degradation rate. The differences in scaffolds impacted cell morphology and behavior. The 0%-ox.–8%-conc. scaffold conferred a round morphology associated with chondrogenesis. However, hADSCs with a larger spreading area as seen in the 5%-ox.–15%-conc. group were correlated with osteogenesis [89,90]. Kim et al. characterized porcine ADSC morphology and transcriptome in a 2% alginate scaffold that was reconstituted from lyophilized alginate. The pADSCs formed osteogenic nodules and displayed increased expression of ALP by day 4 after exposure to osteogenic media. An early increase in the expression of BGLAP, COL1A1, SPARC, and SPP1 was noticeable by day 2. Cell morphology was rounded in the 3D alginate scaffold contrary to fibroblast-like shape in 2D culture prior to osteogenesis. Alginate stiffness and environment play a role in cell attachment and osteogenesis, since a stiff environment may encourage cells to differentiate into an osteoblast lineage and limit migration [91,92]. Guneta et al. show hADSCs prefer osteogenic differentiation in alginate scaffolds with a stiffness greater than 11.61 kPa [93]. A study conducted by Ghiasi et al. determined the effects of multiple scaffolds, including alginate, on hADSC viability and proliferation. The alginate scaffold was reconstituted by creating an alginate mixture from powder and cells. After 14 days of culturing in a 1.2% *w*/*w* alginate scaffold, hADSCs expressed the pluripotent markers OCT4A, NANOG, and SOX2, indicating the preservation of stemness. Compared to the FG, PLGA, and active and inactive PRP scaffolds, the cell-laden alginate gel displayed the lowest viability and proliferation, which may be due to the lack of integrin binding sites for cells [94,95]. Despite alginate being a commonly used material in tissue engineering due to its biocompatibility and low toxicity, Arg-Gly Asp (RGD) conjugation may often be required for increased cell adhesion [96]. 

##### Collagen

Alkaline phosphatase can be cross-linked onto collagen fibers to improve osteogenic differentiation [97]. A study conducted by Jafary et al. evaluated hADSC differentiation on collagen fiber scaffolds with covalently immobilized alkaline phosphatase. Greater osteocalcin, ALP, and collagen expression were detected on the third day with expression decreasing on the seventh day. The levels of RUNX2 were downregulated compared to the collagen only group on the third day with no significant difference on the seventh day. Expression of TNF-α, an inhibitor gene for osteogenesis, decreased on the third day. Altogether, osteogenesis was improved when ALP was immobilized on collagen scaffolds, with osteogenic gene upregulation and inhibitor gene downregulation indicating the potential of seeding enzymes on cell-laden collagen scaffolds [98]. Geometrically honeycomb collagen scaffolds seeded with hADSCs can induce osteogenesis. Greater Cbfa-1 expression was present in the cells seeded in the collagen scaffold indicating osteogenesis activation. Cell migration was enhanced, as by day 14, the hADSCs were migrating deeper into the collagen scaffold. Additionally, in vivo findings showed positive von Kossa staining and osteocalcin immunostaining indicating the formation of mineralized nodules and bone formation [99]. Furthermore, aligned collagen type I fibers placed in synthetic electrospun PLGA/PCL scaffolds can have effects on cell morphology and migration which has implications on guided bone regeneration. Rat ADSCs had an oval nucleus with directionally oriented bipolar morphology in comparison to the multipolar morphology present in non-aligned fibers. The presence of the collagen fibers increased the expression of osteogenic genes; however aligned collagen fibers were better than the random fibers in osteogenesis [100].

##### Gelatin

Canine ADSCs (cADSC) were used in making gelatin-induced osteogenic cell sheets to observe osteogenic differentiation and proliferation. The addition of gelatin to osteogenic medium results in greater cell proliferation, upregulation of BMP-7, and positive alizarin red staining [101]. A similar study used frozen–thawed gelatin-induced osteogenic cell sheets with cADSCs for in vivo repair in a canine model. Large callus formation was present in all groups, but better cortical bone connectivity was present in the gelatin treated groups relative to the untreated ADSCs. These findings indicate the potential of gelatin in fracture repair due to greater ossification and mature bone presence in vivo [102]. Furthermore, Wofford et al. evaluated commercially acquired purified gelatin scaffolds seeded with hADSCs in a rat maxillary alveolar bone defect model. The gelatin scaffold was not cytotoxic and encouraged hADSC growth and attachment post seeding. Expression of OPN and RUNX2 were significantly upregulated with osteogenesis occurring with no adverse effects of the gelatin scaffold. Rats treated with hADSC + Gelatin scaffolds had greater bone formation early on with Masson trichrome staining verifying organized ossification [103]. A recent study conducted by Wang et al. tested the effectiveness of reconstituted gelatin scaffolds with hADSCs. The scaffold was biocompatible, as cell adhesion and proliferation were sustained. Interestingly, OCT4, nestin, and SOX9 were downregulated, while osteocalcin was upregulated more than 2-fold in a 14-day period. The findings suggest gelatin scaffolds emphasize osteogenic differentiation compared to other lineages. Von Kossa and alizarin red staining detected the formation of calcium nodules within 14 days. The scaffold was implanted in a rat calvarial defect and greater bone formation was present relative to the untreated group indicating enhanced osteogenesis with the gelatin scaffold [104]. 

##### Hyaluronic Acid

Aguiari et al. seeded hADSCs onto a hyaluronan-based sponge and observed differentiation into osteogenic, chondrogenic, and adipogenic lineages. In the osteogenic culture, positive ALP activity was detected along with osteonectin, osteopontin, and osteocalcin expression. Markers for adipocyte and chondrocyte differentiation were not. Mineralized bone matrix formation was surrounding the cells indicating osteogenesis in the hyaluronan scaffold [105]. mADSCs in hyaluronan-coated or hyaluronan-supplemented cultures demonstrated greater osteogenic potential along with long term cell preservation. Cell senescence was significantly less in both hyaluronan groups, indicating that hyaluronan suppresses cell senescence for long-term proliferation. Greater calcium deposition was shown in both hyaluronan groups, detected by silver nitrate staining. In addition, osteogenic potential was preserved in passages greater than 5 [106]. Although this experiment was not conducted in a 3D culture, it highlights the potential application of hyaluronan in preserving cell osteogenic differentiation and lifespan for future prospects with ADSCs. Similarly, hADSCs were cultured in hyaluronic acid-derived scaffolds for corneal repair. The ADSCs cultured on the commercially acquired Hystem-HP scaffold had the greatest survivability since the environment promoted cell growth. Cells in the hydrogel scaffold survived long-term up to 10 weeks, indicating the preservative effects of hyaluronan [107]. More experimentation with ADSCs in hyaluronan-based scaffolds should be conducted to further characterize its uses in bone regeneration and osteoblast formation. 

#### 3.1.2. Natural Composite Scaffolds

##### Alginate/Collagen

A study conducted by Yeo et al. fabricated hydrogels with a collagen core seeded with hADSCs with an outer alginate sheath through a custom-made 3D bioprinter. hADSCs in the collagen center were healthy and proliferation increased over a 7-day period significantly compared to alginate-based control scaffolds. Cell viability of hADSCs in the composite was at 91% compared to the 83% in the alginate hADSC control scaffold, showing collagen’s cell-supportive nature. Live/dead fluorescent images of the hADSCs in the composite scaffold showed greater live cells on day 7. In addition, hepatogenic genes ALB and TDO2 were higher in the composite scaffolds indicating potential hepatogenic differentiation. Currently, this may have future use in liver regeneration studies [108]. For bone regeneration, osteogenic genes should be evaluated along with the impact of alginate/collagen concentration on hADSC phenotype in accordance with osteoblast differentiation. 

##### Collagen/Hyaluronic Acid

Xu et al. used ADSCs in reconstituted collagen/hyaluronic acid composites for vocal fold regeneration. ADSC viability a week post-seeding was improved along with greater proliferation which suggests the scaffolds foster ADSC growth [109]. Another study conducted by Amann et al. co-cultured hADSCs with human articular chondrocytes in collagen/hyaluronic acid scaffolds reconstituted via gelation. The greater hyaluronic acid concentration was associated with lower SOX9 expression indicating chondrogenesis inhibition. Metalloproteinase 13 expression increased which is related to matrix remodeling. Lower concentrations of hyaluronan, such as 1%, resulted in SOX9 upregulation and glycosaminoglycan production which is associated with chondrogenesis. In addition, the low O_2_ levels detected in the scaffolds may contribute to improved chondrogenesis. The co-culturing of articular chondrocytes and hADSCs prefer chondrogenesis, but hyaluronan concentration may be modified to prevent chondrogenesis [110]. Hyaluronan concentration in a HA/collagen composite scaffold and osteoblast co-culturing with hADSCs should be investigated for use in bone regeneration. The effects of each biomaterial on ADSCs are summarized in Table 3. 

### 3.2. Bone-Marrow-Derived Stem Cells

#### 3.2.1. Natural Scaffolds

##### Alginate

pBMSCs cultured on a 2% alginate scaffold displayed increased expression of osteogenic genes but had a delayed increase in ALP and BGLAP. The pBMSCs formed osteogenic nodules by day 4 and stained positive for ALP by day 14. ALP presence was detected a few days after the presence of ALP in a 2D culture, indicating the possibility of poor diffusion of differentiation factors or dyes through the alginate hydrogel. Cell morphology was spherical prior to osteoblast differentiation in the 3D culture. pBMSCs exhibited substantial viability in the alginate under osteogenic conditions over the period of differentiation as detected by an MTT assay [91]. A study comparing alginate-CaCl_2_, alginate-CaSO_4_, alginate-gelatin, and alginate-nanocellulose seeded with BMSCs was conducted to determine osteogenic and grafting potential through extrusion 3D bioprinting. There were fewer viable cells in the alginate-CaCl_2_ and alginate-gel scaffold, but these groups had the lowest apoptotic levels. The greatest cell death occurred in the alg-CaSO_4_ and alg-nanocellulose scaffolds after 7 days possibly due to the mechanical properties of the hydrogel limiting nutrient exchange. The alg-CaCl_2_ scaffold displayed the greatest calcium deposition and positive osteocalcin expression indicating it is favorable for osteogenesis. To further evaluate the scaffolds, a scaphoid bone was bioprinted using the alg-CaCl_2_ bioink with BMSCs with viable cells post printing. After 2 weeks, calcification was present with positive alizarin red staining. Despite the increase in calcification, deposition was not homogenous and was focused near the peripheral edges [111]. Future applications of cell-laden bone fabrication should be explored with a focus on vascularization and improved mechanical properties as alginate concentration may impact nutrient and waste diffusion [112].

##### Collagen

Collagen is rich in integrin binding sites, which promotes cell adhesion and can be combined with other materials to offer improved mechanical and biological properties [113]. The addition of type I collagen to a culture can induce osteoblast differentiation in bone marrow stromal cells. This was indicated by upregulation of differentiation markers ALP, Cbfa-1, OCN, and mineralization that was present at three weeks [114]. A study by George, Kuboki, and Miyata created lyophilized honeycomb collagen scaffolds with rBMSCs and determined the differentiation potential into osteoblasts. Greater collagen content and ALP activity was detected on the honeycomb collagen scaffold along with calcium-deficient hydroxyapatite synthesis. The scaffolds contained pore sizes of 200–400 µm and promoted osteogenesis as verified through X-ray diffraction and Von Kossa staining [115]. Apart from type I collagen, type II collagen-coated culture plates with hBMSCs demonstrated significant RUNX2 upregulation along with osteocalcin by day 6, indicating that type II collagen affects calcium deposition. The hBMSCs also expressed upregulation of the type II collagen receptor integrin α2β1 by day 4 during osteogenic differentiation indicating attachment to the type II collagen. In a powder reconstituted HA/TCP scaffold coated with type II collagen, increased mineral deposition and cell calcification were detected relative to the control or type I collagen coated scaffolds. Additionally, denser bone formation indicating mature bone was present during the assessment of segmental bone defect repair in rats. On day 7, there was a significant improvement in walking function for mice treated with the type II collagen-coated HA/TCP and type I collagen-coated HA/TCP scaffold. However, sciatic function index scores were consistently higher for the type II collagen coated scaffold but after day 28 equivalent outcomes were shown in walking function [116]. The use of collagen fibers and BMSCs in biomimetic scaffolds is promising with future applications expanding on bone reconstruction.

##### Gelatin

Mazaki et al. created a lyophilized gelatin-furfurylamine hydrogel photo-crosslinked with Rose Bengal. The hydrogels were seeded with BMSCs, BMP4, and collagen binding domains. Rose Bengal alone is cytotoxic to cells, but the addition of the gelatin-FA hydrogel confers cytoprotecting function as cell viability increased significantly. The scaffolds were implanted into a rabbit osteochondral defect and after 12 weeks the BMSC-laden gelatin-FA hydrogel exhibited cartilage and bone growth similar to the surrounding areas, indicating osteogenesis and chondrogenesis [117]. Another study by Yao et al. synthesized a BMSC-laden lyophilized gelatin hydrogel with horseradish peroxidase and galactose oxidase enzymatic crosslinking in dermal regeneration. The encapsulated BMSCs had greater proliferation and viability which may be due to the less exposure of H_2_O_2_. There was a faster healing time in dermal wounds, but the implications for bone regeneration should be explored, as the presence of reactive oxygen species increases during a fracture [118,119]. 

##### Hyaluronic Acid

Hyaluronic acid in a 3D culture can have positive implications in inducing osteogenesis in BMSCs. Hyaluronan hydrogels synthesized from powder and conjugated with N-cadherin mimetic peptides demonstrate an increase in osteogenic markers in vitro and mineralization both in vitro and in vivo. There is significant upregulation of type I collagen, osteocalcin, ALP, and RUNX2 after 4 days of osteogenic differentiation in the Cad + RGD hyaluronan scaffold. Cell viability is markedly increased due to increased cell adhesion in the RGD peptide conjugated HA scaffold. There is greater mineralization and angiogenesis occurring in the Cad + RGD group, indicating successful bone matrix formation. Micro CT skull reconstruction in rat calvarial defects displayed greater bone formation related to the Cad + RGD group, showing in vivo efficacy in critical size defect repair. Expression of osteocalcin and RUNX2, blood vessel formation, and higher bone volume in the Cad + RGD HA group in vivo signifies increased bone matrix deposition, along with the formation of osteoblasts [120]. A separate study conducted by Cavallo et al. evaluated the use of bone marrow concentrate (BMC) containing stem cells, immune cells, and resident marrow cells in HA scaffolds. Hyaff-11 scaffolds were seeded with the BMC replicating the bone marrow microenvironment to induce osteogenesis to repair osteochondral lesions. The BMC cells were viable post-seeding and proliferated successfully until day 40. Mineralization occurred after day 40 with the presence of dark nodules confirmed via Von Kossa staining. Type I collagen, BSP, and ALP levels increased around the 40-day mark. RUNX2 levels were high at day 0 but decreased until day 52 indicating early bone formation [121]. This indicates hyaluronan has positive implications for early-onset osteogenesis. 

#### 3.2.2. Natural Composite Scaffolds

##### Alginate/Collagen

Perez et al. designed a hydrogel carrier with a collagen–BMSC core and an alginate outer shell for cell delivery. The encapsulated BMSCs had a cell viability of 70% throughout a 21-day period; however, proliferation was persistent for up to 21 days. Cell survival in the carriers depended on the alginate/collagen concentrations, as alginate concentrations that are too high result in hydrogel stiffness and nutrient diffusion limits. There was greater BSP, OPN, and OCN expression in the hydrogel composite carriers compared to the collagen gel by day 14, indicating a greater initial propensity for osteogenesis. The in vivo rat calvarial model had the greatest osteogenesis in the BMSC-encapsulated carrier group which was differentiated for 7 days. The nondifferentiated BMSC carrier group displayed a 25% increase in bone volume but was exceeded by the differentiated carrier group with up to 45% ossification [122]. In conclusion, more experimentation should be conducted on BMSCs in alginate/collagen scaffolds to determine the full effects on osteogenesis in vitro and in vivo.

##### Collagen/Hyaluronic Acid

rBMSCs in collagen/hyaluronic acid scaffolds fabricated by freeze-drying demonstrate early chondrogenesis and drastic upregulation of SOX9 and collagen type II. Cell proliferation increased within 28 days in the collagen/hyaluronan scaffold compared to the collagen-only scaffold. In addition, greater cell infiltration was present in the composite scaffold despite smaller pore size indicating an influence on cellular migration [123]. Additionally, the findings of Zhang et al. demonstrate that the use of collagen/hyaluronic acid seeded with BMSCs are suitable for cartilage tissue engineering [124]. Differentiation of the BMSCs into chondrocytes makes this scaffold ideal for chondrogenesis compared to osteogenesis. The use of strict BMSC-laden collagen/hyaluronic acid hydrogels in bone tissue engineering is scarce and should be further evaluated for bone defect repair [125]. Table 4 summarizes the effects of each biomaterial on BMSCs. 

## 4. Conclusions

Current bioprinting methods are capable of printing complex 3D structures seeded with stem cells for bone tissue engineering; however, they are yet to surpass autologous bone grafts. Extrusion, inkjet, and laser-assisted bioprinting each have their advantages and disadvantages in their use for printing stem cells. Furthermore, the implementation of nanotechnology and functionalized materials are opening new avenues with bioprinting [126,127]. Their implications on cell viability and differentiation should be considered prior to the use of these methods. Mesenchymal stem cells such as adipose-derived and bone-marrow-derived stem cells provide routes for enhancing osteogenesis due to their differentiation ability. When these stem cells are placed into a scaffold, osteogenesis can be induced and cause fracture reparation. BMSCs and ADSCs exhibit similar and different properties in the various scaffolds discussed. Both gravitate towards chondrogenesis in collagen/hyaluronic acid composites and have strong proliferation in alginate/collagen composites and gelatin. Interestingly, BMSCs have the potential to differentiate into a chondrogenic line, as well as an osteogenic one in gelatin, while the studies discussing ADSCs involve enhanced osteogenesis. Overall, both cell types have upregulated osteogenic genes, such as RUNX2, OPN, and OCN, contributing to osteogenesis. Aside from osteogenesis, chondrogenesis may occur, depending on the biomaterials used. Natural polymers can be printed individually or mixed to form composites with cells. These natural scaffolds can enhance differentiation, cell viability, and cell adhesion depending on the polymer’s properties. The biodegradability, biocompatibility, and osteoinductive effects of the desired scaffold should be evaluated prior to use with the stem cells in order to maximize osteogenic potential. In addition, composite scaffolds provide a mix of properties from both biomaterials which can confer better traits than an individual biomaterial. Therefore, the printing method, combination of biomaterials, and type of stem cells must be carefully considered prior to osteogenic differentiation for fracture repair. In summary, while many natural polymers have been used to evaluate BMSCs and ADSCs in vitro and in vivo, more experimentation must be performed to maximize the efficiency of using these cells in biomaterials. Furthermore, the process of fracture repair is dynamic, and investigation should be focused on the interaction between the biomaterials, cells, and the underlying bone tissue in in vivo models. This allows insight into the molecular mechanisms of repair mediated by the biomaterials and cells. Currently, the interactions between 3D bioprinting and cell-laden bioink have been characterized across extrusion, inkjet, and laser-assisted bioprinting with respect to viability, genotoxicity, and migration. However, more studies need to be conducted to further evaluate the impact on osteogenesis between cell-laden biomaterials and 3D bioprinting, as many scaffold compositions need further testing. Between these three methods, careful consideration must be taken when determining the composition of the bioink to generate a scaffold. Only by optimizing this process can experiments create an ideal resulting scaffold to maximize bone regeneration.

## Figures and Tables

**Table 2:** Effects of bioprinting techniques on adipose and bone marrow stem cells.

	Bioprinting Method			
	Extrusion	Inkjet	Laser	References
Adipose Stem Cells	Drop in viability due to shear stress, cells can attach to hydrogels normally and grow, printed monolayers show a higher cell viability, retention of differentiation ability	Favorable cell adhesion depends on the biomaterial, increase in cell proliferation after 24 h, may create incomplete constructs due to printing lower cell densities	Does not initiate differentiation, no effect on proliferation, no significant DNA damage	[40,41,42,43,44,51,52,53,59,60,61,62]
Bone Marrow Stem Cells	Sheer stress may encourage cells into osteoblast lineage, long-term differentiation potential is retained, lower cell viability, cell proliferation increases within 28 days	Cell proliferation and viability affected by higher pressures, medium shear pressure encourages differentiation, unchanged stem cell phenotype post printing, osteogenic differentiation not affected by printing	No changes in phenotype, no significant effect on cell proliferation, high cell viability, no significant genotoxicity or apoptosis occurred	[37,45,46,47,48,55,56,59,65,66,67]

**Table 3:** Adipose stem cells in scaffolds.

Biomaterial	Effects	Bioprinting Method *	References
Alginate	Increased BGLAP, COL1A1, SPP1 expression, round morphology, stiffness may encourage osteoblast differentiation, stemness preservation, low viability	Inkjet	[91,93,94]
Collagen	Osteogenic genes are upregulated with inhibition of TNF-α, enhanced in vivo bone regeneration, multipolar or bipolar morphology	-	[98,99,100]
Gelatin	Good cell proliferation and adhesion, ossification in vivo, enhance osteogenesis, early upregulation of OPN and RUNX2	-	[101,102,103,104]
Hyaluronic Acid	Can cause osteogenic, chondrogenic, or adipogenic differentiation, mineralized matrix formation and calcium deposition, long term cell preservation	-	[105,106,107]
Alginate/Collagen	High viability and cell proliferation	Custom	[108]
Collagen/Hyaluronic Acid	Strong cell proliferation, associated with chondrogenesis, greater hyaluronic concentration inhibits chondrogenesis	-	[109,110]

* If applicable.

**Table 4:** Bone marrow stem cells in scaffolds.

Biomaterial	Effects	Bioprinting Method *	References
Alginate	Early osteogenic nodule formation, poor diffusion of differentiation factors/dyes, spherical morphology, lower cell viability, presence of calcium deposition	Extrusion	[91,111,112]
Collagen	Can induce osteogenic differentiation, mineralization present by 21 days, RUNX2 upregulation, cells bind to integrin proteins	-	[114,115,116]
Gelatin	Cytoprotective function, osteogenesis and chondrogenesis, strong proliferation and cell viability	-	[117,118]
Hyaluronic Acid	Increase in osteogenic markers, blood vessel formation and mineralization in vivo, early bone formation	-	[120,121]
Alginate/Collagen	Lower cell viability but persistent cell proliferation, survival depends on alginate/collagen concentrations, greater BSP, OCN, and OPN expression	-	[122]
Collagen/Hyaluronic Acid	Early chondrogenesis, upregulation of SOX9 and collagen type II, greater cell migration	-	[123]

* If applicable.

## Data Availability

Not Applicable.
